# Strategies to Get Drugs across Bladder Penetrating Barriers for Improving Bladder Cancer Therapy

**DOI:** 10.3390/pharmaceutics13020166

**Published:** 2021-01-27

**Authors:** Shupeng Wang, Shaohua Jin, Qinghai Shu, Song Wu

**Affiliations:** 1 School of Material Science and Engineering, Beijing Institute of Technology, Beijing 100081, China; 3120170538@bit.edu.cn (S.W.); jinshaohua@bit.edu.cn (S.J.); 2 School of Medicine, Shenzhen University, Shenzhen 518000, China

**Keywords:** intravesical treatment, drug instillation, bladder cancer, bladder penetrating barriers, urothelium, bladder mucosa

## Abstract

Bladder cancer is a significant public health concern and social burden due to its high recurrence risk. Intravesical drug instillation is the primary therapy for bladder cancer to prevent recurrence. However, the intravesical drug therapeutic effect is limited by bladder penetrating barriers. The inadequate intravesical treatment might cause the low drug concentration in lesions, resulting in a high recurrence/progression rate of bladder cancer. Many strategies to get drugs across bladder penetrating barriers have been developed to improve intravesical treatment, including physical and chemical methods. This review summarizes the classical and updated literature and presents insights into great therapeutic potential strategies to overcome bladder penetrating barriers for improving the intravesical treatment of bladder cancer.

## 1. Introduction

Bladder cancer is one of the most common urinary system tumors, ranking among the 10 most common malignancies worldwide [1]. Bladder cancer is classified into non-muscle invasive bladder cancer (NMIBC) and muscle invasive bladder cancer (MIBC), the proportions are approximately 70% and 30%, respectively [2]. The occurrence and progression of bladder cancer are heterogeneous, resulting in various clinical outcomes, and nearly 170,000 cases died from this disease every year in the world [3,4,5]. Transurethral resection of bladder tumor (TURBT) is the first-line treatment for bladder cancer. The subsequent intravesical instillation of Bacillus Calmette–Guérin (BCG) and chemotherapeutic drugs are used to prevent cancer recurrence. However, up to 50% of patients still have tumor recurrence within 5 years [6,7]. The reason of bladder cancer recurrence is difficult to explain, which may be related to the existence of cancer stem cells, tumor dissemination, and sporadic tumor micro foci after TURBT [8,9]. On the condition that intravesical instillation cannot effectively kill the residual bladder cancer cells, recurrence may occur. Patients with a high risk of recurrence/progression do poorly on currently recommended therapeutic regimens, so alternative schedules are urgently required [6].

In the last 10 years, high-throughput genome sequencing has revealed genomic characterization of bladder cancer [10], and immune checkpoint inhibitors are applied to the treatment of advanced urothelial carcinoma [11,12]. However, bladder cancer is still a significant public health concern and social burden due to its high recurrence risk, associated with considerable treatment costs [13]. To reduce the potential risk of bladder tumor recurrence and treatment failures, effective therapy still needs enough attention, especially reliable intravesical drug instillation.

## 2. Drugs of Intravesical Treatment

### 2.1. Immunotherapy

Bacillus Calmette–Guérin (BCG) is the live attenuated vaccine form of *Mycobacterium bovis*, which is primarily used against tuberculosis [14,15]. Intravesical BCG to treat bladder cancer is since 1976, BCG can induce immune responses in the bladder mucosa to treat residual tumors after TURBT and prevent the recurrence/progression of bladder cancer, which is the currently recommended therapy [16]. However, intravesical BCG is possible to induce severe stimulus response, even cause dysuria, hematuria, and cystitis [17]. To reduce adverse events of intravesical immunotherapy, the attenuated Salmonella Enterica Typhi Ty21a vaccine-strain (Ty21a) may take place of BCG. The pre-clinical data demonstrates that Ty21a has excellent efficacy and safety. Th1-type immune responses induced by Ty21a are similar to that of BCG, besides, Ty21a is more effective than BCG to anti-tumor capacity in the established bladder cancer model [18].

### 2.2. Chemotherapy

For intravesical chemotherapy, epirubicin, doxorubicin (DOX), mitomycin (MMC), and gemcitabine are the most common agents. In addition to drug species, the intravesical chemotherapeutic effect is also related to urine pH, instillation time, and the concentration of chemotherapy drugs [19]. Multiagent combined intravesical chemotherapy has been proposed in recent years [20,21,22]. Such intravesical multiagent regimens have the potential to offer better efficacy than conventional intravesical chemotherapy, which may also be an alternative to patients who are unresponsive or tolerant to BCG [23,24,25].

### 2.3. Photodynamic Therapy

Intravesical photodynamic therapy is an alternative strategy for bladder cancer. When the photosensitizer is activated by laser light with a specific wavelength, the photosensitizer will react with oxygen to generate singlet oxygen to destroy bladder cancer cells [26,27,28,29]. Commonly used photosensitizers include hematoporphyrin and 5-aminolevulinate [30,31]. Photodynamic therapy has showed encouraging therapeutic effects, but it also leads to toxicity on normal cells. In the future, the antibody-conjugate photosensitizer will be a more target agent for intravesical photodynamic therapy [29].

### 2.4. Gene Therapy

Intravesical gene therapy has achieved satisfactory results for bladder cancer treatment in clinical trials, especially for high-risk BCG-unresponsive NMIBC [32,33,34,35]. CG0070 is a gene therapy based on the oncolytic adenovirus containing a cancer-target promoter and a granulocyte-macrophage colony-stimulating factor (GM-CSF) gene. CG0070 can kill bladder cancer cells through cell lysis based on oncolytic adenovirus and immune-mediated cell killing induced by GM-CSF. The Phase II clinical trial have showed that in high-risk NMIBC patients who are refractory or have relapsed from BCG therapy, intravesical CG0070 results in overall 44% and 30% complete response rates at 6 and 12 months, respectively [33]. Different from CG0070, rAd-IFN/Syn3 gene therapy is based on the non-replicating recombinant adenovirus encoding the interferon alpha-2b (IFN-α2b) gene, and Syn 3 is a transfect enhancer [35]. In the Phase III Study of rAd-IFN/Syn3, more than half of the patients with high-grade NMIBC achieved a complete response at 3 months [36].

## 3. Advantages and Challenges of Intravesical Instillation

Due to the bladder’s unique anatomical characteristics, intravesical therapy is preferred over the oral and intravenous routes for treating bladder cancer [37]. In most cases of oral and intravenous route, only a small fraction of drugs reach desired lesions, owing to loss from systemic metabolism or poor absorption. In contrast, intravesical treatment is a non-invasive drug administration route through a catheter, which is expected to maximize lesions exposure to the therapeutic agent, minimizing or even eliminating systemic side effects.

The efficacy of intravesical instillation depends on effective drug penetration into bladder tumors. However, the low permeability of bladder penetrating barriers limits the drug concentration of bladder tissues. With the excretion of urine, the intravesical drug failed to penetrate the bladder will be diluted or washed out, reducing the effect of intravesical treatment.

## 4. Bladder Penetrating Barriers

**Figure 1:**
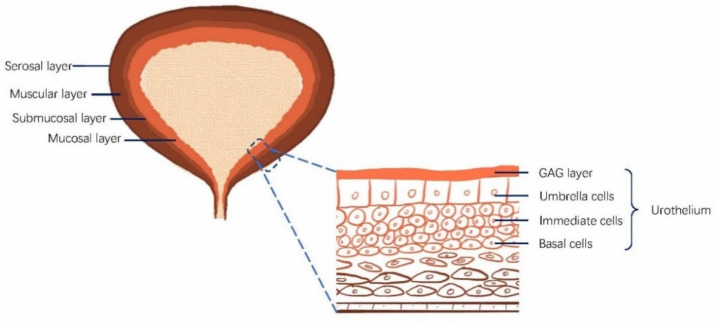
Bladder structure and the urothelium. The urinary bladder walls comprise four layers: the mucosa (urothelium), the submucosal layer, the muscular layer, and the serosal layer. The urothelium includes umbrella cells, intermediate cells, and basal cells. Glycosaminoglycan (GAG) is located at the urothelial luminal surface to form the negatively charged GAG layer, contributing to the bladder barrier function.

The bladder is a spherical hollow organ to store urine and urinate. The bladder wall is composed of several layers, the serosa, muscularis propria, the submucosal layer, and the mucosa (urothelium) [38]. The urothelium is multilayered, consisting of a layer of superficial umbrella cells, several layers of intermediate cells, and a layer of basal cells, as shown in Figure 1. The umbrella cells are connected by tight junctions, which prevent the paracellular diffusion of substances [39,40]. The urothelium is considered impenetrable to the vast majority of all the urine substances present [40,41]. Glycosaminoglycan (GAG) is located at the urothelial luminal surface to form the negatively charged GAG layer, also contributing to urothelial barrier function [42,43,44].

**Figure 2:**
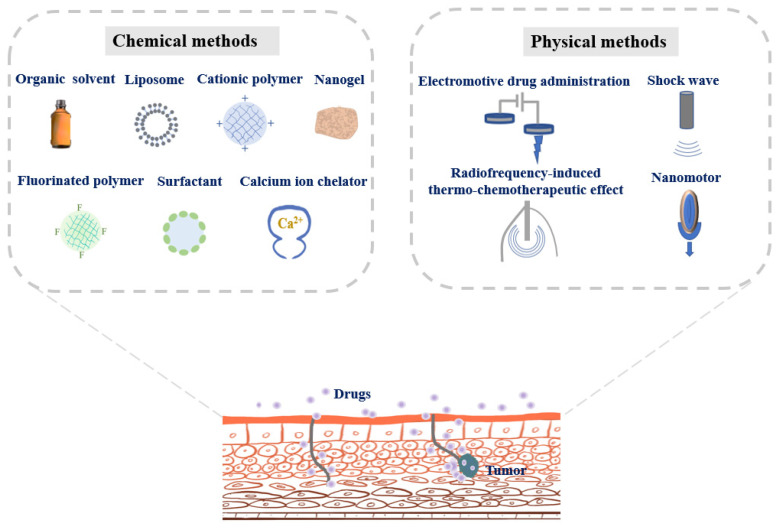
Chemical and physical strategies to get drugs across bladder penetrating barriers for improving bladder cancer therapy.

The distinctive structure of the urothelium with a high transepithelial electrical resistance provides a permeation barrier, also known as the bladder permeability barrier [45,46,47]. The bladder permeability barrier prevents the penetration of bacteria and harmful substances from urine into the blood. Meanwhile, the barrier imposes restrictions on passive diffusion of intravesical drugs. Therefore, opening the bladder permeability barrier is of great significance to effective intravesical instillation to treat bladder cancer. Herein, chemical and physical means to get drugs across bladder penetrating barriers are presented in Figure 2, and the development stage of each strategy has been detailed in Table 1.

## 5. Chemical Methods to Get Drugs across Bladder Penetrating Barriers

### 5.1. Organic Solvents

Dimethylsulfoxide (DMSO) is a dipolar solvent, miscible with lipid and water. DMSO can affect the lipid bilayer, thereby increasing the drug penetration in cytomembrane and biological barriers. DMSO has also been approved by U.S. Food and Drug Administration (FDA) for interstitial cystitis/bladder pain syndrome, which proved DMSO is safe for intravesical instillation [75]. Co-administration of DMSO can promote small molecule drugs across the urothelium [76,77]. Yaman et. found intravesical instillation of epirubicin with DMSO enhanced the epirubicin absorption of the bladder wall, the fluorescence of epirubicin was observed throughout the bladder tumor and in the deeper muscle layers. In contrast, epirubicin’s fluorescence was only seen in the bladder mucosa in the epirubicin without DMSO group [48]. Acetone has been shown to improve BCG attachment to the bladder wall by removing the bladder mucosa [49]. Ramesh et al. reported intravesical ethanol pretreatment enhanced adenovirus-mediated gene transfer in both normal and neoplastic urothelium, indicating ethanol also destroyed the bladder barriers [50].

### 5.2. Cationic Polymers

Polyamidoamine (PAMAM), a highly branched dendrimer, is available for loading a wide range of drug molecules and has shown the penetration capacity into three-dimensional cell spheroids, intestines, and the skin as novel drug carriers [78,79,80]. However, the highly positive charge of PAMAM may cause toxicity to the epithelium, so PAMAM was modified with polyethylene glycol (PEG) to form biocompatible PEG-PAMAM, which could help DOX penetrate deeper into the bladder wall. The amount of DOX within the bladder tissues was also increased after intravesical installation [51].

Chitosan is a cationic polysaccharide with biocompatibility. Positively charged chitosan could facilitate adherence to the negatively charged mucosa, disrupt the biological barrier’s integrity, loosen intercellular tight junctions, and facilitate paracellular drug transport [80,81,82,83]. The surface of poly(lactic-*co*-glycolic acid) (PLGA) nanoparticles (NP) were modified by low molecular weight chitosan, these chitosan-modified NPs demonstrated 10-fold increased uptake in the mouse bladder than unmodified NPs, and chitosan-modified NPs encapsulated survivin siRNA resulted in about 65% reduction in bladder tumor volume [52]. Interleukin-12 (IL-12), a potent inducer of the innate immune system, was co-delivered with the chitosan (CS/IL-12) for intravesical treatment of bladder cancer. In vivo experiments suggested that chitosan enhanced the intravesical delivery of IL-12 to boost the anticancer effect [53].

Poly guanidinium oxanorbornene (PGON) is a positively charged cell-penetrating polymer, the addition of PGON to the surface of PLGA NPs significantly improved bladder penetration of NPs by 10-fold compared to NPs without PGON in the mouse bladder [54].

### 5.3. Fluorinated Polymers

Owing to unique hydrophobic and lipophobic behaviors of fluorocarbon, fluorinated polymers could be used as gene/protein carriers for effective transmembrane penetration [84,85,86]. Previous studies have proven that fluorinated polyethylenimine (F-PEI) could self-assemble with the mastoparan I (MPI) peptide, F-PEI significantly increased mucosal and tumor permeability of MPI in the bladder; therefore, intravesical treatment with MPI/F-PEI NPs resulted in remarkably improved therapeutic responses compared to other controls [55]. Moreover, Li et al. found fluorinated chitosan (FCS) could modulate transepithelial electrical resistance and open tight junctions of uroepithelium by transmission electron microscope, which performed better than PEI in the ability to improve transmucosal and intra-tumoral penetration. Moreover, FCS dramatically enhanced intravesical sonosensitizer conjugated catalase delivery for improving the treatment effectiveness of bladder cancer in vivo [56].

### 5.4. Liposomes

The liposome is an attractive drug delivery carrier with lipid bilayers that resembles the structure of cytomembranes. Maleimide-modified PEGylated (Mal-PEG) liposomes were developed as mucoadhesive vehicles for intravesical therapy of bladder cancer [57]. Mal-PEG liposomes encapsulated fluorescein sodium exhibited greater penetration and retention abilities on bladder mucosal compared to unmodified liposomes. GuhaSarkar et al. have designed an in situ-gelling liposome-in-gel (LP-Gel) system using fluidizing liposomes incorporated into gellan hydrogel [58]. LP-Gel utilizes urine to undergo ion-triggered gelation to form a cross-linked gellan matrix. The system mimics the bladder mucosa, thereby allowing better interaction and adhesion to the bladder wall. After LP-Gel system is instilled into the rat bladder, the ion-triggered gelation adheres to the urothelium, the fluidizing liposomes then penetrate through the urothelial barrier, therefore prolonging drug localization in tumor lesions. Instillation of paclitaxel-loaded LP-Gel showed prolonged drug localization in the bladder at least 7 days, suggesting potential use in clinical practice.

### 5.5. Surfactants

Surfactants are a series of compounds that lower the surface tension, which may act as detergents, emulsifiers, or dispersants. A few surfactants have been approved by FDA to promote penetration through the skin, such as sodium octyl sulfate and sodium laureth sulfate [87,88]. Some studies have also reported that surfactants could enhance urothelial penetration of adenoviruses and chemotherapeutic agents [63,89]. Adenovirus is one of the most promising vectors for gene therapy, the coxsackievirus and adenovirus receptor (CAR) mediates adenoviral attachment and infection by CAR-independent cell entry. However, the GAG layer retards the adenoviral adherence to the CAR of the urothelium, further reducing the therapeutic effect based on adenovirus gene vectors. Some surfactants could disrupt the GAG layer, such as dodecyl-beta-D-maltoside [32], sodium dodecyl sulfate [50], cetylpyridinium chloride [60], and Syn3 (a synthetic polyamide surfactant) [90]. Significantly, the intravesical gene therapy based on recombinant adenovirus with Syn3 (rAd-IFNα/Syn3) has been under clinical trials [63].

Tween 80 (polysorbate 80) is a nonionic surfactant often used in medicines and cosmetics, which has been found to enhance intravesical chemotherapy. The addition of Tween 80 could improve the doxorubicin concentration in the rat bladder wall [61]. The docetaxel solution (Taxotere^®^, DTX-sol) also promotes the docetaxel absorption in the bladder tissue by adding Tween 80. Besides, Brij 98, a polyoxyethylene surfactant, also could as the permeation enhancer for intravesical instillation. The low surface tension of Brij 98-containing nanoemulsions could facilitate cisplatin to permeate across urothelium, resulting in the high concentration and retention ability of cisplatin in the bladder tissues [62].

### 5.6. Calcium Ion Chelators

Calcium ions play an essential role in maintaining cell–cell junctions [91,92,93]. The removal of extracellular calcium in cultured epithelial cells could lead to the opening of intercellular tight junctions to enhance paracellular permeability [94]. Calcium ion chelators have been used in breaking tight junctions of the urothelium and intracellular junctions of bladder tumors. Polycarbophil is a synthetic polymer by the cross-linking of polyacrylic acid with divinyl glycol, which could chelate with extracellular calcium ions, resulting in the opening of cellular tight junctions [64,95]. In the isolated porcine bladder, intravesical polycarbophil (1% *w*/*v*) increased the bladder tissue penetration of pipemidic acid by 4-fold. EDTA (Ethylenediaminetetraacetic acid) is an aminopolycarboxylic acid with the capacity to bind to calcium ions to form a hexadentate chelating agent [38]. Bao et al. have recently developed an innovative EDTA-based nano-platform, which can deprive Ca^2+^ from the intercellular calcium-dependent connexin by EDTA-Ca^2+^ chelation, resulting in the opening of intercellular junctions and the disaggregation of bladder cancer cells [65]. This non-invasive strategy presents excellent clinical prospects for intravesical therapy of bladder cancer.

### 5.7. Nanogels

The intravesical instillation can be further improved by enhancing the drug mucoadhesive capacity, thereby preventing the drug from being washed away during urination. Nanogels are nanosized hydrogels formed by highly crosslinked polymer networks, which can be designed to prolong the retention period to the urothelium and penetrability of drugs toward the bladder wall [96]. The polypeptide nanogel of poly(l-lysine) poly(l-phenylalanine-*co*-l-cystine) (PLL–P(LP-*co*-LC)) was synthesized to deliver 10-hydroxycamptothecin (HCPT) for treating orthotopic bladder cancer [66]. The positive surface charge and amphipathicity gave the nanogel excellent permeation and retention properties in the bladder wall, further boosting antitumor effect toward bladder cancer. In addition, a PEG-modified nanogel of oligoarginine-poly(ethylene glycol)–poly(l-phenylalanine-*co*-l-cystine) (R9-PEG–P(LP-*co*-LC)) was developed for intravesical instillation. Modification of PEG and R9 significantly improved HCPT-nanogel with enhanced mucoadhesiveness, owing to the nonspecific interaction between PEG chains and the urothelium along with the electrostatic interaction between the cationic R9 and the negatively charged bladder mucosa. Moreover, R9 further promoted the permeability of nanogel/HCPT into the bladder wall. The intravesical instillation of nanogel/HCPT remarkably inhibited tumor progression in the orthotopic bladder cancer model [67].

### 5.8. Others

Protamine is a positively charged protein that originated from salmon sperm. Protamine can damage the negatively charged GAG layer of urothelium through electrostatic interaction [97]. It is widely used to make experimental animal models of interstitial cystitis/bladder pain syndrome by inducing the bladder permeability barrier’s dysfunction [47,98]. Hyaluronidase is an endoglycosidase with the ability to hydrolyze hyaluronic acid of the GAG layer to disrupt the integrity of urothelium [99]. Owing to severe damage to the bladder barrier function, protamine and hyaluronidase may not be suitable for intravesical therapy. The dysfunction of bladder penetrating barriers caused by protamine and hyaluronidase will be likely to result in undesirable side effects, such as increased urgency, frequency, and pain during urination.

## 6. Physical Methods to Get Drugs across Bladder Penetrating Barriers

### 6.1. Electromotive Drug Administration

Electromotive drug administration (EMDA) is a widely used device-assisted intravesical therapy, which creates a potential gradient between the bladder wall and the intravesical drug solution under an electric field’s influence [68,100,101]. The electrical field is generated between a catheter electrode placed on the bladder wall’s surface and a cutaneous electrode sticking on the abdomen to aid the transport of drug molecules into tissues [69,102]. EMDA temporarily increases the drug permeability through the bladder barrier by electro-osmosis and electroporation [102]. EMDA increased the MMC uptake in bladder tissues, the MMC concentration was 30-fold higher using EMDA than passive MMC in the urothelium and three-fold higher in the lamina propria and muscularis [103]. In clinical trials, EMDA is considered safe and effective for high-risk NMIBC patients with “BCG refractory”, which could be the salvage treatment for these patients to avoid radical cystectomy [68,69].

### 6.2. Radiofrequency-Induced Thermo-Chemotherapeutic Effect

Radiofrequency-induced thermo-chemotherapeutic effect (RITE) has been developed to improve intravesical chemotherapy, especially for BCG-unresponsive NMIBC [104,105]. The minitype antenna in the catheter with radiofrequency at 915 MHz is directed at the bladder wall. The thermo-energy induced by radiofrequency can effectively penetrate bladder tissue thickness to enhance tissue and cell permeability, so as to improve intravesical drug absorption [102]. Besides, bladder tumors are more susceptible to hyperthermia than normal tissues, and nanotubes on the cancer cell membrane induced by RITE further improve intracellular drug delivery [106,107]. Intravesical 40 mg MMC combined with RITE over 60 min resulted in 10-fold higher MMC concentration in the bladder tumor tissue than intravesical MMC alone [108]. In a systematic analysis, only 26 out of 93 (28.0%) patients had the bladder cancer recurrence in the RITE group, versus 67 out of 99 (67.7%) in the MMC alone group, indicating lower recurrence risk in the RITE group than the MMC only group [70]. Besides, a recent study revealed patients with high-risk NMIBC and unresponsive/intolerant to BCG treatment would most likely benefit from this technology, the 24-month disease-free survival (RFS) was 81.8% in the RITE group, versus 64.8% in the BCG group [71].

### 6.3. Shock Wave

The extracorporeal shock wave has been applied for lithotripsy for decades [109,110]. Recently, low-energy shock wave (LESW) therapy has been reported to increase the cell and tissue permeability without apparent damage, which has been applied to ameliorate tissue ischemia and enhance intracellular delivery [111,112,113]. LESW can overcome the bladder barrier through acoustic pulses, thereby strengthening the drug’s passive diffusion into the bladder wall to increase the local drug concentration. Elkashef et al. has investigated the effect of LESW on enhancing intravesical epirubicin delivery in a rat bladder cancer model. They found LESW enhanced urothelial permeability, and epirubicin concentration was increased by 5.7-fold compared to intravesical epirubicin alone. Moreover, LESW-assisted intravesical epirubicin therapy resulted in less tumor invasion and lower mortality rates [72]. Therefore, LESW may be a feasible device-assisted therapy for treating bladder cancer.

### 6.4. Nanomotors

Compared to passive diffusion, self-propelled nanomotors have shown great potential for applications in the active drug delivery [114,115,116]. Nanomotors with autonomous power are capable of enhancing tissue retention and penetration to overcome biological barriers [117,118]. Tang et al. have fabricated a type of nanometer by asymmetrically modifying platelets with the urease [73]. When these nanomotors are exposed to urea, the urease-mediated enzymatic reaction will produce carbon dioxide and ammonia to form the driving power, which actuates the motor for active movement. Polydopamine shows good biocompatibility and high adhesion property to biological tissues [119,120,121]. Choi et al. developed a polydopamine-based nanomotor, which was surface-functionalized with urease. When intravesical polydopamine-based nanomotors are exposed to the high level of urea in urine, they autonomously and efficiently penetrate deeply into the bladder mucosa and remain for a long period [74]. Nanomotors have opened the door for the active intravesical delivery to overcome bladder penetrating barriers. The enhanced penetration and retention of nanomotors as intravesical drug delivery vehicles will also be used to treat other bladder diseases.

### 6.5. Focal Injury

Griffin et al. found the peptide CGKRK (Cys-Gly-Lys-Arg-Lys) could penetrate mucosal layers following focal mechanical damage to the urothelium [122]. After the focal injury, the intravesical peptide could bind to the entire urothelium, further penetrating the deeper tissues than the control without the injury treatment. Moreover, rhodamine-loaded CGKRK-nanogels can also be effectively delivered to the bladder mucosa after the focal injury. The above results indicated that limited focal injury might be contributed to the entire intravesical delivery.

## 7. Conclusions

Intravesical drug therapy is an indispensable approach for treating bladder cancer. However, due to the existence of bladder penetrating barriers, the drug cannot effectively enter into the lesions after intravesical instillation, inadequate treatment will result in the recurrence of bladder cancer. In order to maximize the therapeutic effect of intravesical drugs, penetration enhanced strategies are essential. In previous studies, various physical and chemical methods have been applied to disrupt the bladder barrier to enhance drug permeability and increase the drug concentration of bladder tumors.

In the future, the efficacy and clinical feasibility need to be a concern to facilitate advanced strategies of penetrating bladder barriers into the clinical practice. The most promising strategies include liposomes, surfactants, nanogels, EMDA, RITE, and low-energy shock wave therapy, some of which have been under clinical trials. Moreover, the combined strategies with different chemical and physical means have great potentials in improving the intravesical therapeutic effect. These developed strategies to get drugs across bladder penetrating barriers will be also used for intravesical therapy of other bladder diseases, such as overactive bladder, cystitis, etc.

## Figures and Tables

**Table 1:** The development stage of each strategy to get drugs across bladder penetrating barriers for improving bladder cancer therapy.

	Strategy	Preclinical Development	Under Clinical Trials
Chemical methods	Organic solvents	DMSO/epirubicin [48] Acetone/BCG [49] Ethanol/adenovirus [50]	
	Cationic polymers	PEG-PAMAM/DOX [51] Chitosan-PLGA NPs [52] Chitosan/IL-12 [53] PGON-PLGA NPs [54]	
	Fluorinated polymers	Fluorinated polyethylenimine/peptide [55] Fluorinated chitosan/sonosensitizer [56]	
	Liposomes	Maleimide-modified PEGylated liposomes [57] In situ-gelling liposome-in-gel/paclitaxel [58]	Proliposomal paclitaxel [59]
	Surfactants	Sodium dodecyl sulfate [50] Cetylpyridinium chloride [60] Tween 80/doxorubicin [61] Brij 98-containing nanoemulsions/cisplatin [62]	rAd-IFN/Syn3 [63] CG0070/dodecyl-beta-D-maltoside [32]
	Calcium ion chelators	Polycarbophil [64] EDTA-based nano-platform [65]	
	Nanogels	(PLL–P(LP-*co*-LC)) nanogel [66] R9-PEG–P(LP-*co*-LC) nanogel [67]	
Physical methods	EMDA		EMDA/MMC [68,69]
	RITE		RITE/MMC [70,71]
	Shock wave	Low-energy shock wave therapy [72]	
	Nanomotors	Asymmetrically modified platelets [73] Polydopamine-based nanomotors [74]	
